# Reproducibility and quality assurance in MRI

**DOI:** 10.1007/s10334-025-01271-1

**Published:** 2025-06-17

**Authors:** Tony Stöcker, Kathryn E. Keenan, Florian Knoll, Nikos Priovoulos, Martin Uecker, Maxim Zaitsev

**Affiliations:** 1 MR Physics, German Center for Neurodegenerative Diseases (DZNE), Bonn, Germany; 2 National Institute of Standards and Technology, Boulder, CO USA; 3 Department Artificial Intelligence in Biomedical Engineering, Friedrich-Alexander-Universität Erlangen-Nürnberg, Erlangen, Germany; 4 Spinoza Center for Neuroimaging, Amsterdam, The Netherlands; 5 Institute of Biomedical Imaging, Graz University of Technology, Graz, Austria; 6 Division of Medical Physics, Department of Diagnostic and Interventional Radiology, Faculty of Medicine, University Medical Center Freiburg, University of Freiburg, Freiburg, Germany

## Introduction

### Challenges in reproducible research

The importance of reproducibility in science has been recognized for centuries. The philosopher Karl Popper once stated, “single occurrences that cannot be reproduced are of no significance to science” [1]. It is reproducibility that distinguishes science from pseudoscience. However, over the past two decades, concerns have increasingly been raised about the reproducibility of scientific studies. Reasons for this include factors such as variability in data collection and analysis, small sample sizes, incomplete reporting of methods, and a lack of standardization of studies. This can lead to inconsistent results and difficulties in comparing or replicating findings [2, 3]. Since then, awareness of the problem has grown, and the global interest of scientists in reproducible research has increased significantly. These encouraging developments have also taken place in the field of medical imaging, as the contributions to this special issue impressively demonstrate. 

### The importance of reproducibility in MRI

MRI technology plays a pivotal role in medical diagnostics and research by providing detailed images of the internal structures of the body, allowing for the diagnosis and monitoring of various diseases, as well as the evaluation of treatment efficacy. While many clinical MRI exams are subjectively evaluated by experienced radiologists, there is an increasing need for more transparent MRI with reproducible and predictable outcomes, e.g., for quantification or automated analysis. On one hand answering medical imaging research questions may benefit from using accurate, precise and reproducible quantitative MRI (qMRI) measurements [4]. Additionally, disease and treatment monitoring in clinical routine is based on the assumption that the outcomes of an examination are reproducible, e.g., any observed significant variation results from disease or treatment progression and not from variations in the measurement process. And finally, the increasing reliance on automated image post-processing, e.g., whole-body organ segmentation [5], requires reliable imaging input free of device or site bias. For these reasons, reproducibility plays an important role in MRI. To improve reproducibility, there is a growing need for dedicated quality assurance (QA) and quality control (QC) procedures. Such procedures quantify experimental stability, detect outliers, minimize variability of outcome measures and thereby improve the significance of final results.

### Definitions

Before we delve deeper into the various topics of reproducibility and quality assurance in MRI, some definitions might be helpful. In addition to Reproducibility, also *Repeatability* and *Replicability* are frequently mentioned in scientific contexts. All terms refer to reobtaining the same (or very similar) scientific results but under different conditions, which are however not uniformly defined across research fields. In computer science, there is consensus on the definition of the “three Rs” in terms of who conducted the research and under which conditions [6]:

- *Repeatability*: same team, same experimental setup
- *Reproducibility*: different team, same experimental setup
- *Replicability*: different team, different experimental setup

These definitions largely coincide with the use of the terms in the contributions to this special issue. For example, the importance of making all information (code and data) available so that other research teams can *reproduce* the results is emphasized several times. However, the above definitions are not sufficient in all cases: the RSNA-QIBA working group defined reproducibility as the set of conditions that involves different locations, operators, or measurement systems [7]. In this issue, Dupuis et al. discuss the repeatability of qMRI measures after a scanner upgrade [8], which means that they investigated the sometimes unavoidable situation of *“same team, different experimental setup”*, which is especially important for longitudinal studies. Therefore, definitions may change slightly depending on the context, and readers should always carefully check whether their own understanding of the terms matches the authors’ intention. The terms QA and QC are simpler and self-explanatory: while QA refers to a proactive action aiming in preventing errors in subsequent data collection, QC is the reactive action to quantify such errors in the data of interest. Finally, these errors may have different origins resulting in limited precision or accuracy. The precision describes the statistical error in the data, and the accuracy quantifies the acquisition bias. In other words, accuracy measures how close results are to the true or best known value, typically acquired with a different experimental setup, and precision measures how close results are to one another, typically acquired on the same setup by the same team and in the same setting. While the precision can be estimated in test–retest experiments (repeatability), the accuracy is often unknown and harder to predict as the true value is usually unknown. Since changes in the experimental setup (e.g. changes of the sequence parameters) may change the accuracy of a particular method, it is a major source of limited reproducibility (or replicability, in the strict sense of the definitions above). In the context of MRI research that relies on clinical whole-body scanners, the second and the third Rs (reproducibility and replicability) often become intermixed due to the reliance on the closed-source vendor software, making it impossible for the research teams to verify all aspects of the experimental setup.

### Purpose of the special issue

There are many reasons that can limit the reproducibility in MRI. One major cause results from the countless possibilities to encode biophysical tissue properties with MRI. Due to the versatility of the MRI measurement process, the physical model for a specific application is in many cases incomplete. Violations of the underlying assumptions introduce a measurement bias. (For instance, the spatial encoding process always contributes some diffusion weighting, which is typically not accounted for in the models for qMRI parameter mapping.) This is the core of MRI research: improving or even developing entirely new physical models that either more accurately describe the measurement process or improve it. New insights could have enormous implications, enabling more realistic and powerful methods for future medical imaging applications. This is *not* the main topic of this special issue. Instead, it addresses the *seemingly* less-exciting issue of limited accuracy or precision due to poorly defined examination conditions. This is an implementation issue. There are a huge number of different possibilities to set up a specific experiment. Once a principle setup is chosen, there are still countless ways to parameterize the complex workflows of MRI data acquisition, reconstruction, and analysis. Additionally, all workflows depend on the given hardware and software environments, and any changes to those may bias the results. Finally, operator-dependent variability may be present if automation is insufficient or in certain cases not possible. Thus, reproducibility (or replicability) in the plethora of MRI applications is a fundamental and difficult problem that may have been overlooked for too long. Fortunately, the advances in reproducible MRI research over the last years have changed that. This special issue addresses the best practices, makes readers aware of existing methods and tools, and identifies current issues and unmet needs, all to improve reproducibility and replicability in MRI. In this context, it is worth mentioning that openness is a necessary prerequisite for reproducibility: to reproduce the research results of others, all information must be available. Therefore, the concepts of “reproducible research” and “open science” are closely linked. Another special issue of this journal will be published later this year focusing on the aspects of open science. Nevertheless, openness is also fundamental to this special issue.

## Strategies for improving reproducibility in MRI

### Method standardization

In this special issue, the question of standardization of MR exams across sites and between vendors is introduced in the review article “Metrology for MRI: the field you’ve never heard of” by Hall et al. [9]. The goal of metrology, the science of measurement, is to ensure consistency in measurements made in different places, at different times, and by different methods. There are examples in MRI where principles of metrology are followed, for example in the determination of SAR guidelines and patient safety, as well as the construction and use of phantoms. However, these are exceptions, and, in contrast to other fields in healthcare like radiotherapy where planning and procedures are tightly controlled, a corresponding metrological framework does not (yet) exist for MRI. It may come as a surprise to many researchers who are used to performing experimental measurements that according to the user manual, an MRI scanner is often not considered to be a measurement device [10]. The goal of an MRI procedure that is performed at a hospital is not to perform a quantitative measurement, but to generate images that are interpreted visually by human experts. This is particularly evident in the development of quantitative MRI. Despite its promise and after decades of research, it is still not used regularly in clinical practice. As the authors point out, the key limitation of current quantitative MRI is reproducibility. Reproducibility relies on a framework that provides reassurances about uncertainty of a particular method, procedures for calibration, and guidance for manufacturers. A key aspect of reproducibility in MRI is the standardization of the complete acquisition and reconstruction pipeline. The review paper by Tamir et al. makes a compelling argument why all details in acquisition and reconstruction, including pre- and post-processing, need to be meticulously described to make advanced computational MRI experiments reproducible [11]. It goes beyond explaining potential issues, but offers a practical cookbook for reproducible MRI based on open tools. The paper by Karakuzu et al. addresses similar questions with a specific focus on quantitative imaging biomarkers [12]. It highlights the importance of standardized and open workflows and offers practical guidelines based on portable and modularized processing pipelines. The Pulseq-CEST Library described by Liebeskind et al. then presents a comprehensive toolbox to address portability and reproducibility for CEST MRI, combining vendor-neutral acquisition, simulation, and evaluation in an open framework [13]. Another example of standardization in MRI protocols and data analysis pipelines in this special issue is in quantitative susceptibility mapping (QSM), presented by Fuchs et al. [14]. Since it is impossible to define an absolute reference for reconstructed susceptibility values, the mean susceptibility within a certain anatomical area is generally used as reference. The authors demonstrate that the choice of the anatomical area that is used as the reference has an impact on the statistical significance of clinical findings. In addition to the introduction of an approach for a non-anatomical reference region based on R2*, the authors recommend the inclusion of reference region values in publications to ensure reproducibility of the results.

### MRI repeatability studies

As mentioned above, the precision of MRI measures can be determined in repeatability studies. This is important for estimating effect sizes and thus the statistical power required for confirming or rejecting a specific hypothesis. Such power analyses are crucial to avoid conducting statistically underpowered studies and thus the publication of non-reproducible results [15]. A nice and positive example of such a repeatability study is given in this issue by Senn et al., who investigated brain tissue R1 dispersion in 20 patients with small vessel disease using field-cycling imaging (FCI) at 0.2 Tesla [16]. The results convincingly show that R1 dispersion significantly differs between healthy and affected tissue and, moreover, it was shown that these results are highly repeatable. However, which metric should one choose to quantify repeatability? There are many possibilities, as shown by Cherukara and Shmueli in this issue. They investigated different repeatability metrics for QSM in head and neck regions obtained from test–retest experiments in ten healthy volunteers [17]. Only moderate repeatability was observed, which additionally strongly depends on the region of interest and the QSM reconstruction method; for the latter, six representative state-of-the-art techniques were utilized. The results suggest that QSM acquisition and reconstruction pipelines should be carefully evaluated before applying them in clinical studies. This is especially important, given that the technique is seen as a very promising tool for the investigation of neurological diseases and especially neurodegeneration [18]. A strong tissue-specific variation of repeatability was also reported by Wang et al. in this issue, who performed test–retest qMRI in the prostate of cancer patients and healthy volunteers [19]. These examples demonstrate that the road from the discovery of a new medical imaging technique to its establishment as a meaningful imaging biomarker is a long and winding one. Repeatability studies are essential along this path.

### Quality assurance and quality control

Many MRI researchers and the majority of clinical sites tend to rely on the QA implemented by the device vendor, that is more or less regularly performed by the service team as a part of the regular device maintenance. However, these potentially turn out to be ineffective for major software or hardware updates, or for larger studies requiring pooling data from multiple centers or scanners from different vendors; this is especially so for studies not based on product features, but on research protocols and experimental pulse sequences and image reconstruction algorithms instead. This calls for establishment of community-driven QA tools. QC of the imaging outcomes is mostly done manually these days by the technician of a radiologist manually reviewing the images while the subject is still on the table and if necessary repeating the scans, e.g. in cases of excessive motion artifacts. The major confounding factor of such practice is the major variability of the quality assessment based on the personal preferences and training as well as the attention span given to this task based on the current work load. Therefore there is a major need for automated and objective image quality assessment, especially due to the increased prevalence of non-linear dictionary-based or AI-driven image reconstruction algorithms, where artifacts are getting increasingly demanding for humans to discern. In this special issue two papers concern themselves with establishing phantom-based QA procedures to either insure the comparability of functional or anatomical brain MRI [20] or establish fast and user-friendly quality assessment procedures for quantitative MRI [21]. A rather different and very comprehensive take on quality assurance is presented in the review paper by Kraff and May, where they summarize QA measures established over years in a consortium of numerous ultrahigh field (UHF) sites, that eventually allowed the consortium to achieve comparable imaging outcomes both in phantoms and in vivo [22]. In contrast, the paper of White et al. [23] focuses on establishing robust image quality metrics. Image reconstructions in MR augmented by artificial intelligence (AI) are becoming increasingly widespread, with the outcomes of the image reconstructions being critically dependent on the model training and parameter tuning of such algorithms. In their manuscript the authors attempt to counteract future challenges to AI reconstructions such as long-term image quality drift, for instance due to software updates, by introducing and validating robust automated image quality metrics. Finally, another paper from the present special issue takes a rather composite approach to QA and QC [24], by introducing a combination of robust experimental setups including mechanical ventilation and trigger timing optimization, as well as data QC during the fitting process. All these measures allow for more reliable liver perfusion measurements based on arterial spin labeling in small animal applications.

### Knowing the ground truth: MRI phantoms

Since the beginning of MRI the community has adopted the use of MRI phantoms for verification and validation of novel methods [25–29]. These phantoms are also powerful for addressing these challenges in reproducibility. For example, in this special issue, Pasini et al. present the results of a multi-center and multi-vendor validation study in two parts for ADC measurement [30] and T1 and T2 measurements [31]. The phantoms are used to demonstrate reproducibility and replicability: is it possible to implement the same or similar protocols for quantitative MRI measurements across sites and vendors? It is necessary to establish this before any multi-site, multi-vendor clinical study of quantitative MRI measurands. One challenge, however, is that these phantoms can be expensive. That is addressed by the work of Yusuff et al. [32] who created a cost-effective 3D-printed MRI phantom. While a home-built phantom can introduce its own variability, the shared CAD file can enable multiple groups to create the same geometric structure. As we look for reproducible science to expand, it is important to make phantoms themselves more accessible.

## Conclusion

Improving reproducibility, quality assurance, and quality control in MRI is a prerequisite for increasing the value of MRI in medical imaging, both in clinical science and in routine diagnostic applications. Therefore, reproducibility has become an active area of research in MRI method development—from hardware to acquisition and reconstruction to analysis. Consequently, the contributions to this special issue cover many different technical aspects of reproducibility in MRI. However, due to the myriad applications and diverse implementation possibilities of MRI, striving for reproducibility remains a challenging task. Therefore, it must become and remain a top priority in our field. Ensuring the robust reproducibility of our methods should be highly valued by the research community and should be treated as being equally important as novel developments. Encouraging, incentivizing and rewarding reproducibility should become a pillar of MRI research, which will require a substantial shift in mind setting from the entire community, in particular, from decision-makers such as principal investigators as well as scientific reviewers and editors. To improve reproducibility and quality assurance in MRI, it is also necessary to develop, continuously update, and disseminate best practices and guidelines for method standardization. Here, openness is crucial, that is, sharing source code, implementation details, and collected data as openly as possible, because reproducing measurement results requires all details of the measurement pipeline. This aspect is repeatedly addressed in several papers of this special issue and is particularly emphasized in the review articles [9, 11, 12, 22]. Reproducibility in MRI benefits significantly from open collaboration and communication between researchers, clinicians, and industry partners. Consistent continuation of these efforts will contribute to more efficient and higher quality MRI research and thus ultimately to better patient outcomes. 
